# Fluorescence Analysis: From Sensing to Imaging

**DOI:** 10.1155/2018/2654127

**Published:** 2018-08-01

**Authors:** Subhankar Singha, Dokyoung Kim, Sankarprasad Bhuniya, Tushar Kumeria

**Affiliations:** ^1^Department of Chemistry, School of Molecular Science (BK21PLUS), Pohang University of Science and Technology (POSTECH), 77 Cheongam-Ro, Nam-Gu, Pohang, Gyeongbuk 37673, Republic of Korea; ^2^Department of Anatomy and Neurobiology, Center for Converging Humanities, College of Medicine, Kyung Hee University, 26 Kyungheedae-Ro, Dongdaemun-Gu, Seoul 02447, Republic of Korea; ^3^Department of Chemical Engineering and Materials Science, Amrita Centre for Industrial Research and Innovation, Amrita School of Engineering, Amrita Vishwa Vidyapeetham, Coimbatore 64112, India; ^4^School of Pharmacy, University of Queensland, 20 Cornwall Street, Woolloongabba, Brisbane, QLD 4102, Australia

Fluorescence sensing and imaging combined with fluorescence microscopy technology has revolutionized human ability to study and visualize complex life phenomena at the molecular level to understand the cellular events with least ambiguity. Being a simple, fast, direct detection system with real-time monitoring capability, fluorescence sensing is highly demanding for *in vitro* as well as *in vivo* analysis in the recent time. In this special issue, we aim to create a platform for introducing the recent advances in the field of fluorescence-based analysis and imaging techniques.

The promising features of fluorescence analysis in turn have inspired a quest for novel fluorescent materials/fluorophores as well as fluorescence probes/sensors with various applications in imaging. In this respect, development of fluorescent materials based on quantum dots has attracted the current research. Importantly, due to advantageous features of lower cytotoxicity, organic quantum dots are preferred over inorganic ones. In this issue, Zhang et al. report a facile, green, and high-output synthesis of highly fluorescent nitrogen-doped carbon quantum dots which show strong blue fluorescence along with high photostability and excellent biocompatibility. The carbon dots further find the utility as a fluorescence probe for cell imaging as well as for detection of Fe^3+^ in serum.

Due to superiority of two-photon excitation-based imaging techniques, especially in the field of medical sciences, development of novel two-photon fluorophores is of highest priority. Kim and coworkers introduce a novel two-photon excitable fluorophore-benzo[*g*]coumarin and summarize the photophysical properties and synthetic methods along with the promising applications for bioimaging and sensing of biologically important species using benzo[*g*]coumarin analogues. An advanced application of two-photon intravital fluorescence microscopy is further demonstrated by Hyun and coworkers to report the sensing of vascular permeability from the inflamed vessel of live animals. The investigation shows how blood vessel is ruptured and vascular leakage occurs during acute inflammation in correlation with neutrophil infiltration at the subvascular level under inflammatory condition in the cremaster muscle of mice. Compared to the previously reported methods, this intravital imaging method helps to measure more accurate vascular leakage in terms of time and location *in vivo*.

As the current research on fluorescence sensing and imaging is majorly focused on disease diagnosis, accordingly development of diagnostic imaging agents for disease biomarkers is also important. Park et al. develop a pyridazine-based fluorescent probe targeting amyloid-beta (A*β*) peptides, the neuropathological hallmarks of Alzheimer's disease (AD). Besides the key features of selective detection and imaging of A*β* plaques through strong fluorescence enhancement, the reasonable hydrophobic nature for blood-brain barrier (BBB) penetration would be helpful to use the probe as a promising imaging agent for AD diagnosis in the near future.

Biomolecule labeling is a common trend, in the recent time, to visualize and track the various biological processes. In the course of labeling, it is essential to separate the nonconjugated residues from the molecules conjugated with labels. Through comparing the average intensity of fluorescence, Kim et al. successfully demonstrate a dielectrophoresis-based separation method between the unlabeled protein (unreacted) and the labeled one with polystyrene beads, onto a single electrode platform having two different sizes of microholes. This research is also scientifically appealing for enhancing the performance of fluorescent sensors.

In conclusion, this special issue covers the broad area of fluorescence analysis starting from the development of fluorescent materials to the applications for fluorescence sensing and imaging, thus attracting the attention from the various research areas including Chemistry, Biology, Materials, and Polymers. Moreover, the research articles containing the original research results and the review articles share the current state of the art of this fundamental research area among the readers of this journal.

*Subhankar Singha* *Dokyoung Kim* *Sankarprasad Bhuniya* *Tushar Kumeria*

